# Comparative genomic analysis of clinical *Enterococcus faecalis* distinguishes strains isolated from the bladder

**DOI:** 10.1186/s12864-023-09818-z

**Published:** 2023-12-07

**Authors:** Baylie R. Hochstedler-Kramer, Adriana Ene, Catherine Putonti, Alan J. Wolfe

**Affiliations:** 1 Department of Microbiology and Immunology, Stritch School of Medicine, Loyola University Chicago, Maywood, 60153 IL USA; 2 Bioinformatics Program, Loyola University Chicago, Chicago, 60660 IL USA; 3 Department of Biology, Loyola University Chicago, Chicago, 60660 IL USA

**Keywords:** *Enterococcus faecalis* genomics, Bladder microbiome, Comparative pangenomics, Niche adaptation

## Abstract

**Background:**

*Enterococcus faecalis* is the most commonly isolated enterococcal species in clinical infection. This bacterium is notorious for its ability to share genetic content within and outside of its species. With this increased proficiency for horizontal gene transfer, tremendous genomic diversity within this species has been identified. Many researchers have hypothesized *E. faecalis* exhibits niche adaptation to establish infections or colonize various parts of the human body. Here, we hypothesize that *E. faecalis* strains isolated from the human bladder will carry unique genomic content compared to clinical strains isolated from other sources.

**Results:**

This analysis includes comparison of 111 *E. faecalis* genomes isolated from bladder, urogenital, blood, and fecal samples. Phylogenomic comparison shows no association between isolation source and lineage; however, accessory genome comparison differentiates blood and bladder genomes. Further gene enrichment analysis identifies gene functions, virulence factors, antibiotic resistance genes, and plasmid-associated genes that are enriched or rare in bladder genomes compared to urogenital, blood, and fecal genomes. Using these findings as training data and 682 publicly available genomes as test data, machine learning classifiers successfully distinguished between bladder and non-bladder strains with high accuracy. Genes identified as important for this differentiation were often related to transposable elements and phage, including 3 prophage species found almost exclusively in bladder and urogenital genomes.

**Conclusions:**

*E. faecalis* strains isolated from the bladder contain unique genomic content when compared to strains isolated from other body sites. This genomic diversity is most likely due to horizontal gene transfer, as evidenced by lack of phylogenomic clustering and enrichment of transposable elements and prophages. Investigation into how these enriched genes influence host-microbe interactions may elucidate gene functions required for successful bladder colonization and disease establishment.

**Supplementary Information:**

The online version contains supplementary material available at 10.1186/s12864-023-09818-z.

## Background

Within 30 years of being recognized as a distinct taxon, *Enterococcus* species gained notoriety for their dichotomous role as ubiquitous commensal gut organisms and opportunistic pathogens. The contrasting lifestyles of this genus are largely attributed to its capacity for adapting to new environments and substantial genomic plasticity [1]. Enterococci are adept at transferring genetic material to bacterial species within and outside of its own genus [2, 3] and have greatly contributed to the spread of antibiotic resistance worldwide, most notably via the vancomycin resistance cassette [4]. Due to intrinsic and acquired resistance mechanisms, multi-drug resistant Enterococci are now regarded as a major public health concern.

*Enterococcus faecalis*, the most prevalent clinical enterococcal species, is associated with chronic and recurrent hospital-acquired infections at diverse body sites [5–8]. This species is also frequently isolated from medical device infections, such as catheter-associated urinary tract infections (UTI) [9]. Furthermore, *E. faecalis*is commonly implicated in polymicrobial infections and can increase virulence of other organisms in these infections [10]. The pathogenic potential of *E. faecalis* is most often studied in the context of these hospital-acquired infections, which are usually thought to occur via fecal contamination of wounds or medical devices [11]; however, recent studies have shown that UTI often precedes bacteremia [12].

*E. faecalis* is also associated with community-acquired UTI and severe bladder infection phenotypes. Recent studies show an increased prevalence of *E. faecalis* in patients with chronic and recurrent UTI, defined as 3+ UTI within one year [13–15]. Despite this association, clinics often underreport and/or fail to detect *E. faecalis* due to poor urine culture methods and reporting schemes. Standard urine culture, originally designed to diagnose *Escherichia coli*in pyelonephritis, biases results towards fast growing, highly abundant, Gram-negative uropathogenic species [16]. As the goal of this method is to diagnose UTI by identifying a monoculture of a pathogenic organism, potential polymicrobial infections are often reported as mixed growth or contamination [17]. Relative to the more sensitive expanded quantitative urine culture (EQUC) method, the standard urine culture method has been shown to detect *E. faecalis* less than 50% of the time in some populations [15]. This underreporting and lack of isolation has limited researchers’ ability to investigate behaviors and genetic components important for persistence in the unique bladder environment.

The discovery of the bladder microbiome raises the possibility that the lower urinary tract, which includes the bladder and urethra, serves as an endogenous reservoir for *E. faecalis*. This environmental niche is hypothesized to be comparatively harsh and nutrient-limited compared to the gut, leading researchers to question if bladder colonization and persistence may only be accomplished by strains harboring or expressing specific genetic factors [18]. Past studies have highlighted the genomic variation between environmental and clinical enterococcal strains but have failed to detect similarly strong trends in clinical strains isolated from diverse body sites [19, 20]. This could be due to comparatively low sample sizes and limited publicly available, sufficiently detailed metadata. For example, many researchers fail to distinguish between microbiota isolated from voided urine, which samples the entire lower urogenital tract (bladder, urethra, and vulvovaginal skin) [21] versus the bladder, which can be sampled directly via transurethral catheterization [22].

In this study, we conducted comparative genomic analyses of 111 whole genomic sequences of clinical *E. faecalis* strains isolated from various body sites, including bladder, urogenital, blood, and fecal isolates. While phylogenomic comparison does not identify distinct lineages based on niche, whole genomic comparison shows significant clustering of blood and bladder strains. Functional enrichment analysis identified clusters of orthologous groups (COGs) and KEGG pathway genes that are significantly associated with strains from each isolation source when compared to bladder strains. Using these enriched genes as training data and 682 publicly available genomes as test data, machine learning algorithms were successfully able to distinguish between bladder and non-bladder strains with high accuracy. This analysis identified mobile genetic elements, specifically phage genes and transposable elements, as being extremely important for accurately identifying bladder genomes. Together, these data highlight the importance of comprehensive genomic comparison, possibly elucidating horizontal gene transfer events that aid in *E. faecalis* bladder niche adaptation.

## Results

This study utilizes whole genome sequences previously obtained from 66 *E. faecalis* strains isolated from the urogenital tract (Supplementary Table 1). Most of these isolates are true bladder isolates (*n* = 36) as they were cultured from catheterized urine specimens, which directly samples the urinary bladder. The remaining, categorized as urogenital isolates (*n* = 30), were cultured from voided urine (*n* = 16) or swab specimens from other urogenital sites (*n* = 13) including the urethra, peri-urethra, and vagina. All bladder and urogenital isolates were obtained via EQUC from various patient populations, such that they were evenly distributed among asymptomatic study participants and patients seeking care for lower urinary tract symptoms (LUTS, Table 1).

**Table 1:** General characteristics of genomes utilized for comparative analysis

	Bladder *N* = 36	Urogenital *N* = 30	Blood *N* = 19	Fecal *N* = 26	*p*-value
LUTS Presence, N (%)	27 (75%)	18 (60%)	NA	NA	0.229
Median Genome Length (bp)	2980141.5	2935212	3005248.5	2961441	0.42
Median Number of Contigs	36.5	36	48.5	5.5	0.48
Median GC Content (%)	37.34	37.32	37.37	37.36	0.62
Median Number of Genes	2949	2892.5	2971.5	2911	0.48

As *E. faecalis* is a known commensal gut organism associated with sepsis in hospital settings, we aimed to compare whole genomes of bladder and urogenital strains with those of previously published fecal (*n* = 26) and blood (*n* = 19) strains (Supplementary Table 1). These isolates, obtained from larger clinical studies, were selected based on genome quality and presence of detailed metadata (e.g., mode of collection), as well as use in previous publications.

### Clinical *Enterococcus faecalis* strains from diverse body niches do not represent distinct lineages but differ by genomic content

**Fig. 1:**
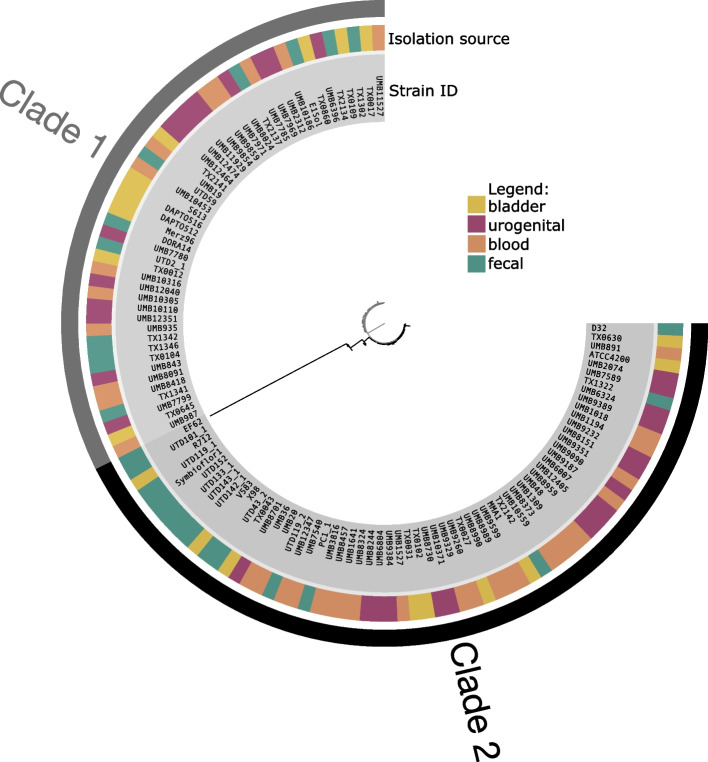
*Enterococcus faecalis* strains isolated from diverse body sites do not represent distinct lineages

General characteristics of these 111 genomes are listed in Supplementary Table 1. The genomes do not differ by genome length (*p* = 0.42), number of contigs (*p* = 0.48), GC content (*p* = 0.62), or number of genes (*p* = 0.48). Amino acid sequences of all ribosomal genes were compared to construct a phylogenomic tree (Fig. 1). This analysis produces two distinct clades, denoted as Clade 1 (gray, *n* = 48) and Clade 2 (black, *n* = 63). Association between clade and strain isolation source was tested for statistical significance. While Clade 2 contains the most bladder strains (36%, *n* = 23), there was no significant association between bladder (*p* = 0.40), urogenital (*p* = 0.82), fecal (*p* = 1), or blood (*p* = 0.51) strains for either clade. Furthermore, each isolation source included several multilocus sequence types (MLST) (bladder = 12, urogenital = 14, blood = 13, and fecal = 17), with 15 MLST types found in two or more anatomical sites.

**Fig. 2:**
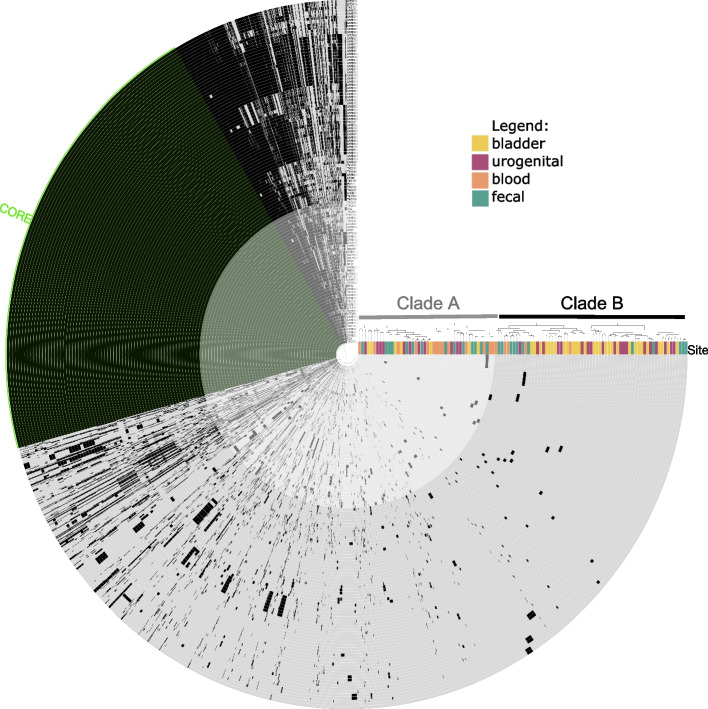
Pangenome analysis shows presence of strain-specific genes. Gene presence is denoted by a black bar for each individual strain, represented by a circular bar graph. Sorting based on gene presence and absence is displayed in the dendrogram

Next, we identified the core- and pan- genomes of this collection of *E. faecalis* strains. The pangenome of all 111 strains consisted of 7228 unique genes, whereas the single-copy core genome (highlighted in green) included only 2007 genes (Fig. 2). The 5221 genes of the accessory genome appear to occur at varying prevalence between the strains. Due to the large accessory genome, the pangenome was then used to cluster genomes based on their gene content. From this sorting, the genomes separate into two new clades: Clade A (gray, *n* = 46) and Clade B (black, *n* = 65), as shown in the dendrogram of Fig. 2. Clade A mostly consists of blood (30%, *n* = 14) and fecal (30%, *n* = 14) strains, whereas bladder strains are disproportionately found in Clade B (45%, *n* = 29). Association between clade and isolation source was again tested for statistical significance; blood strains were significantly more likely to belong to Clade A (frequency = 74%, *p* = 0.004), while bladder strains were significantly more likely to belong to Clade B (frequency = 81%, *p* = 0.002). Clade A and B were equally likely to possess urogenital (*p* = 0.69) and fecal (*p* = 0.22) strains.

### Functional enrichment analysis reveals differentially present genes in bladder strains

**Fig. 3:**
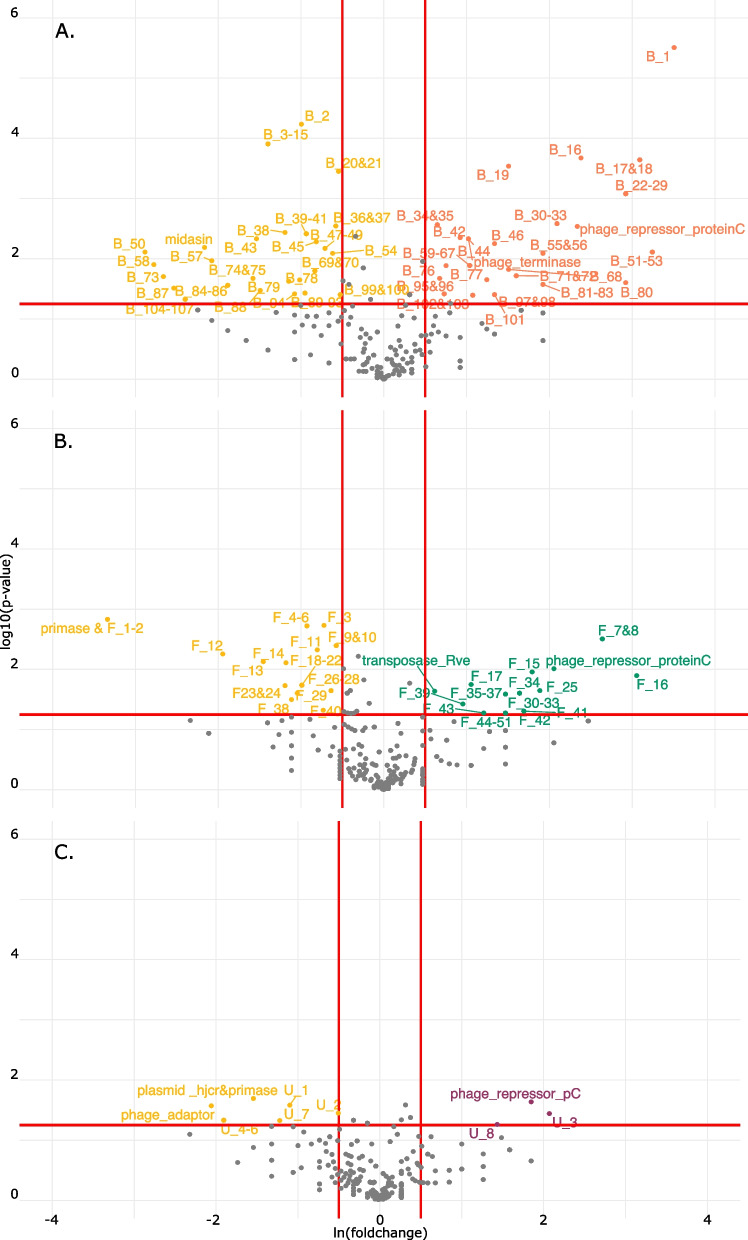
Functional enrichment analysis identifies COG and KEGG hits significantly enriched in bladder strains. Genes significantly enriched in bladder strains (yellow, **A**-**C**) fall above the 1.25 y-intercept and to the left of the -0.5 x-intercept. Genes enriched in blood (coral, **A**), fecal (green, **B**), and urogenital (purple, **C**) fall above the 1.25 y-intercept and to the right of the 0.5 x-intercept. Gene labels containing gene names are shared between functional enrichment and machine learning analyses

**Fig. 4:**
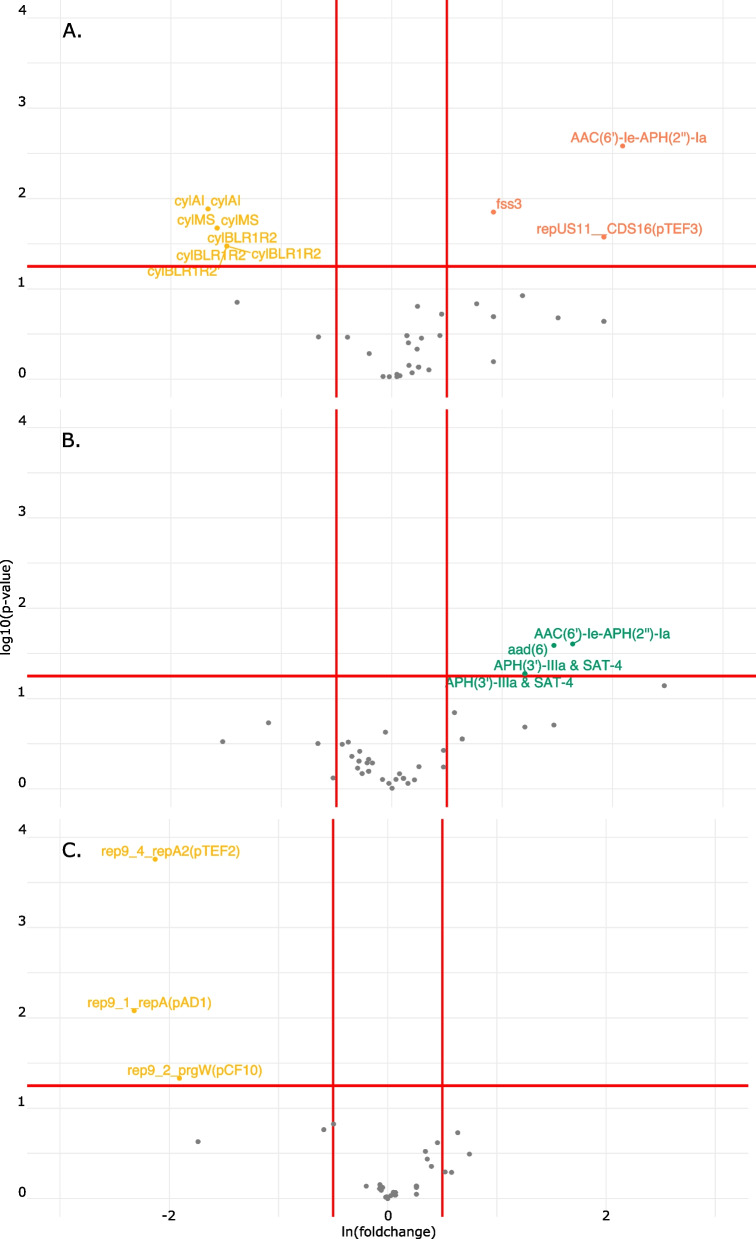
Functional enrichment identifies ABRicate hits significantly enriched in bladder strains. Genes significantly enriched in bladder strains (yellow, **A**-**C**) fall above the 1.25 y-intercept and to the left of the -0.5 x-intercept. Genes enriched in blood (coral, **A**), fecal (green, **B**), and urogenital (purple, **C**) fall above the 1.25 y-intercept and to the right of the 0.5 x-intercept

We then performed functional enrichment analysis to compare genomes based on the functionalities encoded. In short, prevalence of gene hits was compared between bladder strains and blood, fecal, and urogenital strains individually. Genes were considered significantly enriched in a group if the *p*-value was less than 0.05. Genes were considered “unique” to a group if the fold-change value was greater than ± 10. All significantly enriched and unique genes are listed in Supplementary Table 2, and significantly enriched genes are visualized using volcano plots (Figs. 3 and 4). In total, this approach identified 340 significantly enriched and unique genes. Most of these genes were identified by the COG (*n* = 219) and KEGG databases (*n* = 77), whereas genes commonly used to compare clinical enterococcal isolates, including targeted searches for virulence factors, antibiotic resistance genes, and plasmids via ABRicate, account for only 13% of these hits (*n* = 44) (Supplementary Table 2). Of the identified enriched genes, the most represented COG categories are "Defense mechanisms” or “Mobilome: prophages, transposons” (25%, *n* = 55) and “General functional prediction only” or “Function unknown” (21%, *n* = 46).

The comparison between blood and bladder strains accounts for over half of the total significantly enriched genes identified (*n* = 183) with the majority of these genes enriched in blood strains (*n* = 93, Figs. 3A and 4A). Blood strains are significantly more likely to possess diverse antibiotic resistance mechanisms, including the vancomycin resistance cassette. The enterococcal cytoylsin operon and CRISPR genes are significantly enriched in bladder isolates compared to blood isolates. Between bladder and fecal genomes, 92 genes are differentially present and 69 of these genes are enriched in fecal isolates (Figs. 3B and 4B). Comparison between bladder and urogenital strains account for the fewest genes (*n* = 60), with most (*n* = 35) being enriched in urogenital isolates (Figs. 3C and 4C).

### Machine learning correctly distinguishes between bladder and non-bladder strains

Using machine learning, we tested if the 111-genome data set examined in depth here could be used to predict the isolation source of other *E. faecalis* genomes, signifying that *E. faecalis* strains undergo niche adaptations. The results of the COG, KEGG, and ABRicate queries were used as the training data to identify features (genes) for predicting the source *of E. faecalis* genomes in our test data set (Supplemental Table 3), which included representatives isolated from the bladder (*n* = 25), urogenital tract (*n* = 69), gastrointestinal tract/fecal (*n* = 481), and blood (*n* = 107). These testing data was subsampled such that each category was equally represented (see Methods). First, we tested classifiers for their ability to distinguish between bladder and non-bladder isolates. The KNN classifier had an accuracy of 0.7120, the Random Forest classifier had an accuracy of 0.8913, and the CatBoost classifier had an accuracy of 0.8913 with an F1 score of 0.4118. All three classifiers had a greater accuracy than the dummy classifier (accuracy = 0.4783, F1 score. = 0.2000). Second, we tested the same three classifiers for their ability to distinguish between genomes isolated from the bladder, urogenital tract, gastrointestinal tract/fecal, and blood. The KNN classifier had an accuracy of 0.2446, the Random Forest classifier had an accuracy of 0.3424, and the CatBoost classifier had an accuracy of 0.3152 with an F1 score of 0.2252. While all three classifiers had an accuracy greater than the dummy classifier (accuracy = 0.2228, F1 score = 0.2252), they do not perform as well as when just two categories were considered.

Given that the CatBoost classifiers for both the one-hot encoding (bladder vs. non-bladder) and multi-class encoding (4 sites) outperformed the KNN and Random Forest classifiers, we next examined the CatBoost classifier's features (genes) with the greatest weight, i.e., the most significant genes to predict the source of a genome (Supplementary Table 4). Many of the genes with an importance value of over 0.5% (*n* = 44) were annotated with protein products related to phages and transposable elements (34%, *n* = 15).

Prophages are prevalent among the 111 genomes; 105 of the genomes contain one or more prophage sequences predicted to be intact. These 105 strains contain one of 33 different prophage species. Many of the phage-associated genes identified by the CatBoost classifier were found in one of three Enterococcal prophage sequences (*n* = 17, Supplementary Fig. 1). These three prophage sequences most closely resemble (1) EfsC1 EF62phi (Cluster 7: query coverage = 100%, percent identity = 99.99%), (2) SEsuP-1 (Cluster 6: query coverage = 67%, percent identity = 99.96%), and (3) SEsuP-1 (Cluster 34: query coverage = 37%, percent identity = 99.12%). These three prophages are prevalent among bladder urogenital strains (Supplementary Table 5) and account for 32 hits with a cumulative importance weight of 14.9%.

## Discussion

In this study, we hypothesized *E. faecalis* strains isolated from the bladder would contain distinct genomic content possibly contributing to their success in the harsh bladder environment. To address this question, we identified differentially enriched genes contributing to niche adaptation by comparing the genomic content of newly sequenced bladder and urogenital *E. faecalis* strains with well-characterized blood and fecal strains. This research represents the largest-scale genomic description of true bladder *E. faecalis* strains to date. This is in comparison to those studies that investigated strains from voided urine, which have the potential to originate anywhere along the urogenital tract, including vulvovaginal skin or fecal contamination. This genomic description coupled with the finding that bladder strains comprise distinct genomic content reveals novel research paths that may allow researchers to decipher host-microbe interactions unique to the bladder environment or certain bladder disease states.

While addressing our hypothesis, we found that clinical *E. faecalis* strains isolated from the four investigated body niches did not originate from distinct lineages, as evidenced by lack of clustering upon comparison of ribosomal genes. This finding echoes previous reports that phylogenomic clustering fails to distinguish between *E. faecalis* isolated from various infection types [19]. However, niche specialization is observed when comparing whole genomic content. The core genome identified in this set of isolates included 2007 genes. A previous study analyzing 2026 *E. faecalis* genomes reported a core genome of similar size (*n*= 2068) and found the pangenome was closed [23]. This suggests a good representation of genomic diversity in this 111-genome set. Clustering strains based on presence of accessory genes revealed bladder and blood strains were statistically distinct, whereas urogenital and fecal strains were dispersed evenly throughout the dendrogram (Fig. 2). Further analysis of the accessory genes identified significantly enriched gene functions, including virulence factors and antibiotic resistance genes, between the various genome subsets. The majority of differentially enriched genes were found when comparing bladder and blood strains, echoing the previous result of significant differential clading.

Although successful with bladder and blood strains, pangenomic analysis failed to substantially distinguish between urogenital and fecal strains. This could be due to considerable hypothesized microbiota crossover between niches. First, relatively few significantly enriched genes were identified between bladder and urogenital strains (*n*= 60). It is highly likely that several urogenital strains could have originated in the bladder or the intestinal tract, as many were cultured from voided urine, which very often contains vulvovaginal and fecal contaminants [21]. Also, fecal contamination is considered a common prelude to blood and bladder infections [24], possibly contributing to overlap between genomes from these sites. These confounding factors likely also underly the conclusions from the machine learning analyses. Despite multiple classifiers distinguishing bladder vs non-bladder strains with high success, the same classifiers did only slightly better than chance when attempting to predict the niche of isolation between the four sites.

Lastly, genes related to mobile genetic elements were also the most prevalent COG function identified in the functional enrichment analysis and machine learning analyses identified genes related to prophage and transposable elements as important for distinguishing between bladder and non-bladder strains. It is well-documented that *Enterococcus* species possess great capacity for horizontal gene transfer, and recent studies have highlighted the importance of the mobilome to genetic variation in clinical isolates [23, 25]. Notably, many of the “important” genes were traced to prophage that were preferentially present in bladder and urogenital isolates. These phage clusters most closely resemble the Enterococcal phages EfsC1 EF62phi and SEsuP-1 (Supplementary Fig. 1 and Supplementary Table 4). The enrichment of these prophage in bladder and urogenital strains could be a product of prolonged exposure to the unique phageome found in the urobiome [26]; nevertheless, the implications of these prophage sequences are unknown. Previously, the Serror group assessed bacterial behavior in the V583 blood isolate upon sequential deletion of prophage sequences, finding that various prophages play a direct role in virulence phenotypes [27]. The potential impact of the three identified prophages on bladder persistence or colonization has yet to be investigated.

Overall, the observed significant differences between blood and bladder strains suggests that certain genomic content makes *E. faecalis* strains more successful in these unique environments. When considering the gastrointestinal tract as an endogenous source of *E. faecalis*, the equal distribution of urogenital and fecal strains in accessory genome clading leads us to hypothesize that the bladder or blood environments select for strains with specialized and distinct genomic content. Our data further suggests it is possible that strains that successfully establish blood infections more often originate from gastrointestinal or urogenital sources as opposed to ascending infection of true bladder strains. However, this study lacks the appropriate sampling to test these hypotheses, as these analyses would require analysis of *E. faecalis* strains present in all four anatomical sites within in the same individual. Regardless, evidence presented in this study prompts further investigation of invasion processes and mechanisms underlying *E. faecalis* specialization within diverse human niches.

## Conclusions

Genomic comparison of clinical *E. faecalis* isolates shows evidence of unique genomic signatures in true bladder strains. Many of the genes important for this distinction were attributed to the presence of prophages found almost exclusively in bladder and urogenital strains. These data demonstrate niche adaptability of *E. faecalis* within the human body. Ultimately, identification of genes enriched in bladder isolates will prompt new research into gene functions unique to or important for bladder colonization and disease establishment.

## Methods

### Collection of isolates

A total of 111 *E. faecalis* genomes were included in this study. Genomes of strains isolated from blood (*n* = 19) and fecal (*n*= 26) samples were retrieved from NCBI GenBank. Genomes of 66 strains collected from genitourinary tract samples were sequenced in a previous study by our lab (BioProjects PRJNA316969, PRJNA970254, and PRJNA1024931). These genomes include 36 bladder urine strains that were isolated via transurethral catheterization, which directly samples the bladder [22]. Genomes from the remaining 30 strains were classified as “urogenital” as they were obtained from swabs of the distal urethra and vulvovaginal skin or from voided urine, which samples the entire lower urinary tract. The MLST for the 111 *E. faecalis*strains was determined using PubMLST [28]. Information regarding these strains can be found in Supplementary Table 1.

### Phylogenetic analysis and comparative genomics

Phylogenetic analysis was performed in Anvi’o [29], using amino acid sequences of single copy core and all ribosomal genes. The resulting phylogeny was subsequently loaded into and rooted in FigTree [30]. The Anvi’o pangenomic workflow was then used to define the core and pan genomes. Genomes were annotated using COG and KEGG databases. Using the pangenome, isolates were sorted based on whole genomic content based on presence or absence of gene clusters. The association between resulting phylogenomic and pangenomic clades and isolation source was tested for significance by conducting chi square or Fisher’s exact tests, when appropriate.

Functional enrichment analysis was performed using various databases. First, the Anvi’o functional enrichment command [31] was used to determine enrichment of COG and KEGG functions based on isolation source. This method provided a comprehensive, untargeted comparison of genetic content amongst this selection of isolates. While this program computes *p*-values for each differentially present gene, it also outputs presence-absence tables for each gene cluster analyzed. These tables were used to sort differential genes based on enrichment scores to prevent false positives caused by rare genes. Next, sequences were queried for known virulence factors (Virulence Factor Database) [32], antibiotic resistance genes (Comprehensive Antibiotic Resistance Database [CARD]) [33], and mobile genetic elements (PlasmidFinder) [34] using ABRicate [35]. To identify significantly enriched genes, the prevalence of individual gene hits was compared between bladder strains and blood, fecal, and urogenital strains individually. The fold change between bladder and individual strain groups was calculated for each gene hit, and statistical significance was determined using chi square or Fisher’s exact tests, when appropriate. The fold change and raw *p*-values were then used to create volcano plots for each comparison.

### Machine learning for niche prediction

Machine learning analyses were conducted in Python using scikit-learn [36], CatBoost [37], and Pandas. Three classifiers were tested, K-Nearest Neighbor (KNN) and Random Forest through scikit-learn and CatBoost; CatBoost uses gradient boosting on decision trees. Default settings were used for both the KNN and Random Forest classifiers. The training data for all three classifiers was the 111-genome set examined in depth previously. Training data was selected from publicly available data. All publicly available genome assemblies of *E. faecalis* were downloaded from NCBI’s Assembly database. For each genome, the source of isolation was identified either from the metadata of the assembly record, BioSample record, or BioProject record or from associated literature. In the event that the isolation source could not be determined, or it was not from one of the four sites of interest (bladder, urogenital tract, gastrointestinal tract/fecal, blood), the genome was removed from our testing data set. Supplemental Table 3 lists the genomes included in our testing data set.

Two different scenarios were considered: bladder vs. non-bladder (one-hot encoding) and distinguishing between the 4 isolation sources (bladder, urogenital tract, gastrointestinal tract/fecal, blood) (multi-class label). 500 iterations were performed for both the one-hot encoding and multi-class label tests with the CatBoost classifier. For the one-hot encoding, we additionally used L2 regularization to avoid overfitting the model. Because our training data set included more gastrointestinal tract/fecal and blood samples than bladder or urogenital, we randomly subsampled the gastrointestinal tract/fecal and blood genomes such that the testing data set consisted of ~ 25% from each of the four categories. A dummy classifier (strategy = ‘uniform’) was also tested to ascertain the accuracy and F1 score expected by chance.

### Prophage prediction

The 111 *E. faecalis*genomes were examined for the presence of prophage sequences using PHASTER via the API [38]. Results were parsed using Python. The intact prophage sequences were clustered to identify instances of the same phage species in multiple genomes. Clustering was conducted using usearch (v11) [39] with the following parameters: cluster_fast and id = 0.7. A representative sequence from the 3 largest clusters was queried against the nr/nt database (exclusive to viral sequences) using megablast.

### Supplementary Information

**Additional file 1: Supplemental Table 1.** Genome information for phylo- and pan-genomic analysis.

**Additional file 2: Supplemental Table 2.** All significantly enriched genes detected by databases

**Additional file 3: Supplemental Table 3.** Genomes used in machine learning test dataset

**Additional file 4: Supplemental Table 4.** Importance of gene hits for machine learning distinguishment of bladder vs non-bladder genomes.

**Additional file 5: Supplemental Figure 1.** Comparison of phage genomic content.

**Additional file 6: Supplemental Table 5.** Phage clusters associated with important gene hits are enriched in bladder and urogenital genomes.

## Data Availability

The datasets analyzed during the current study (whole genome sequences for bladder and urogenital strains) are available in the BioProjects PRJNA316969 in the NCBI repository, (https://www.ncbi.nlm.nih.gov/bioproject/?term=PRJNA316969 and PRJNA970254 https://www.ncbi.nlm.nih.gov/bioproject/?term=PRJNA970254).
