# Supplementary figures and images for: Comparative genomic analysis of clinical Enterococcus faecalis distinguishes strains isolated from the bladder
Source: BMC Genomics. 2023 Dec 7;24:752. doi: 10.1186/s12864-023-09818-z (PMC10701997; doi:10.1186/s12864-023-09818-z)

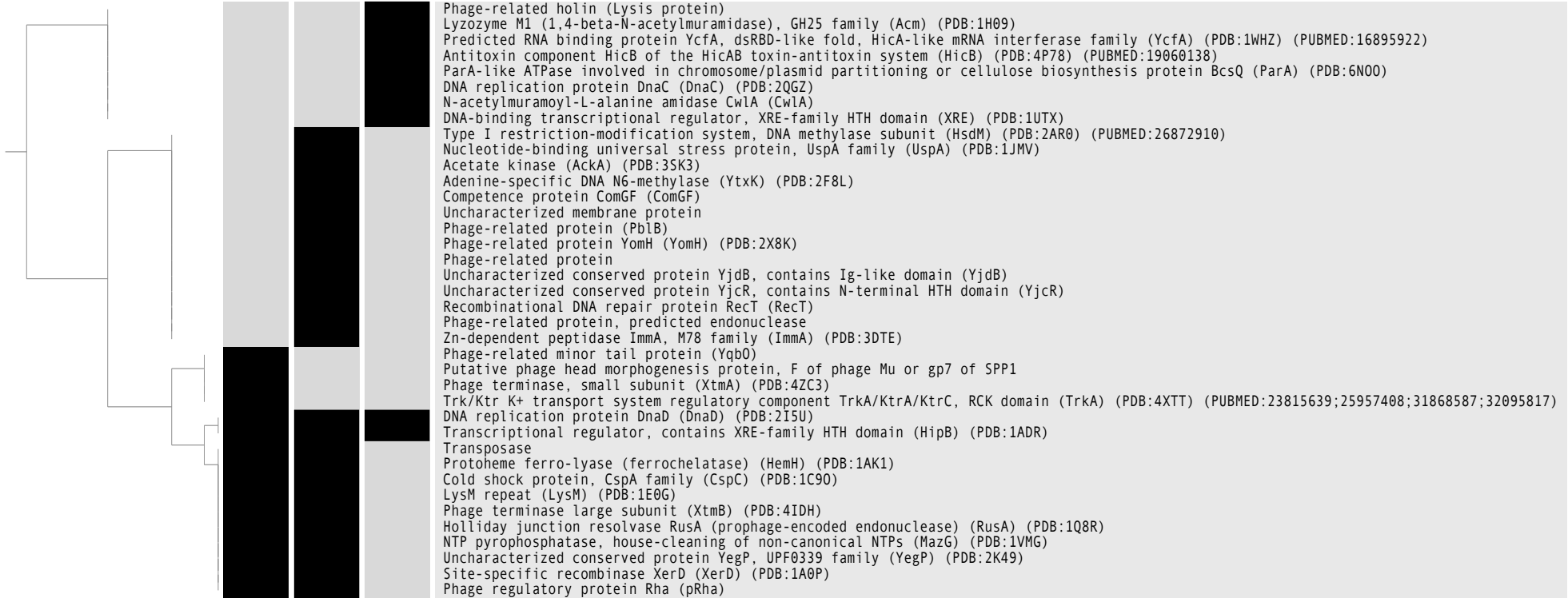

Cluster34:EfsC1EF62phi

Cluster6:SEsuP-1

Cluster7:SEsuP-1

COG20 FUNCTION

Supplement: Supplementary file 5 — Additional file 5: Supplemental Figure 1. Comparison of phage genomic content. [file 12864_2023_9818_MOESM5_ESM.pdf]
