# Supplementary material for: Comparative genomic analysis of clinical Enterococcus faecalis distinguishes strains isolated from the bladder
Source: BMC Genomics. 2023 Dec 7;24:752. doi: 10.1186/s12864-023-09818-z (PMC10701997; doi:10.1186/s12864-023-09818-z)
Supplement: Supplementary file 6 — Additional file 6: Supplemental Table 5. Phage clusters associated with important gene hits are enriched in bladder and urogenital genomes. [file 12864_2023_9818_MOESM6_ESM.pdf]

Supplemental Table 5. Phage clusters associated with important gene hits are enriched in bladder and urogenital genomes.

Most similar characterized phage	Enterococcus phage1 EfsC1 EF62phi Cluster7	Enterococcus phage SEsuP-1 Cluster6	Enterococcus phage SEsuP-1 Cluster34
Query coverage, percent identity	100%, 99.99%	67%, 99.96%	37%, 99.12%
Bladder genome occurrence (n)	9	4	5
Urogenital genome occurrence (n)	5	9	2
Fecal genome occurrence (n)	1	2	1
Blood genome occurrence (n)	0	0	2
Total occurrence (n)	15	15	10
Associated gene hits[a] (n, percent combined importance)	10, 5.78%	24, 9.89%	10, 4.73%

[a]Gene hits identified as important to machine learning algorithm for niche of origin determination.
